# Prevalence and risk of sexual violence victimization among mental health service users: a systematic review and meta-analyses

**DOI:** 10.1007/s00127-024-02656-8

**Published:** 2024-04-03

**Authors:** Anjuli Kaul, Laura Connell-Jones, Sharli Anne Paphitis, Sian Oram

**Affiliations:** Health Service and Population Research Department, Institute of Psychiatry, Psychology & Neuroscience, King’s College London, De Crespigny Park, London, SE5 8AF UK

**Keywords:** Sexual violence, Service user, Trauma, Inpatients, Outpatients

## Abstract

**Purpose:**

People with mental disorders are more likely to experience sexual violence than the general population, but little is known about the prevalence of sexual violence in people who use psychiatric services. This paper aims to estimate the prevalence and odds of sexual violence victimisation within mental health services by gender and mental health setting (i.e. inpatient, outpatient and mixed settings).

**Methods:**

This study is a systematic review and meta-analysis (PROSPERO registration number: CRD4201810019). Three databases (Medline, Embase, PsychINFO) were searched and citation tracking, and reference screening of included studies was conducted. Studies were included if the prevalence and/or risk of sexual violence in psychiatric service users were reported or calculable across the past year or adult lifetime. The methodological quality of included studies was assessed. A random effects meta-analyses was conducted to estimate odds ratios and pooled prevalence estimates of sexual violence in different mental health settings.

**Results:**

Twenty-six studies were included encompassing 197,194 participants. The meta-analyses revealed high pooled prevalence estimates and increased odds of sexual violence victimisation in male and female psychiatric service users compared to non-psychiatric service users.

**Conclusions:**

Mental health practitioners should be trained to respond effectively to disclosures of sexual violence, particularly from these vulnerable groups. Future sexual violence interventions should consider mental health as a treatment outcome.

**Supplementary Information:**

The online version contains supplementary material available at 10.1007/s00127-024-02656-8.

## Introduction

Sexual violence is a global public health issue defined by the World Health Organisation as “any sexual act, attempt to obtain a sexual act, unwanted sexual comments or advances, or acts to traffic or otherwise directed against a person’s sexuality using coercion, by any person regardless of their relationship to the victim, in any setting, including but not limited to home and work” [1]. The prevalence of sexual violence varies between countries. For instance, in the UK 20% of women and 4% of men aged 16 years and over experienced sexual assault since the age of 16 [2]. In Australia 17% of women and 4.3% of men aged 18 years and over had experienced sexual assault since the age of 15 [3].

Sexual violence has been identified as a major determinant of mental ill health, with victims of sexual violence exhibiting higher rates of depressive disorders, PTSD, general anxiety disorders and eating disorders [4]. Mental health services could therefore potentially act as an effective target site for identifying and responding to victims of sexual violence. To confirm this and to shape effective service provision we conducted a systematic review and meta-analysis to estimate the prevalence and relative risk of past year and adult lifetime sexual violence in men and women who use psychiatric services, disaggregated by the type of mental health service setting they are in contact with.

## Methods

### Search strategy

The protocol for this review is registered with the PROSPERO International prospective register of systematic reviews database (available from: https://www.crd.york.ac.uk/prospero/display_record.php?RecordID=100191); registration number CRD4201810019. The search strategy followed MOOSE and PRISMA guidelines [5] (see Online Resource 1). Searches used a combination of Medical Subject Headings (MeSH) and text words (see Online Resource 2) to search the 3 electronic databases Medline, Embase, and PsychINFO from their dates of inception to 18th July 2022. Reference list screening and forward citation tracking using Web of Science and Google Scholar was conducted for all included studies.

### Selection criteria

Studies were eligible for inclusion if they: (a) included male or female mental health service users; (b) included participants aged 18 years or older; (b) presented the results of peer-reviewed research based on the following study designs: experimental studies (e.g. randomised controlled trials, non-randomised controlled trials, parallel group studies) which measure sexual violence at baseline; before and after studies which measure sexual violence at baseline; interrupted time series studies; cohort studies; case–control studies, and cross-sectional studies; (c) measured the prevalence of adult lifetime and/or past year sexual violence victimisation, and/or the relative risk of sexual violence victimisation (i.e., odds ratios, prevalence ratios, rate ratios), or collected data from which these statistics could be calculated; d) were written using the English language.

This review used the World Health Organisation definition of sexual violence defined above [1]. Participants met the criteria for adult lifetime sexual violence if their experiences occurred age 16 years or older. Studies investigating childhood sexual violence victimisation i.e. people who experienced sexual violence before the age of 16, were excluded. Mental health service use was defined as being in contact with psychiatric inpatient, outpatient, community, perinatal, liaison, addiction, veteran psychiatric inpatient, and forensic mental health services.

Where multiple eligible papers from the same study were identified, only the paper reporting the largest number of participants with data of relevance to the review’s objectives were included. There were no restrictions on the geographic location of the included studies.

### Data extraction

LCJ, AZ and AK conducted searches at separate time points between 31st May 2018 and 18th July 2022. The results of the database searches were exported to Rayaan [6] and duplicates were removed. Titles and abstracts were screened against the inclusion criteria. If it was unclear whether a reference met the inclusion criteria, it was taken forward to the next screening stage. A second reviewer (SO) independently screened a random sample of 20% of the records until 95% agreement was reached between the first and second reviewer. Any disagreements were discussed and resolved.

Full text copies of all papers included at the abstract screening stage were screened against the inclusion criteria (LCJ, AK and AZ). A second reviewer (SO) independently screened 20% of the full text papers until 95% agreement was reached between the first and second reviewer. Any disagreements were discussed and resolved. Reference list screening and forward citation tracking was conducted on all included papers and eligible papers were added to the review.

Data from all included papers were extracted into a standardised form (LCJ and AK) including bibliographic information, study design, psychiatric service setting, sample characteristics, instruments used to ascertain participant mental health and occurrences of sexual violence, raw prevalence data of lifetime/past year sexual violence and odds ratios. Where possible gender-specific prevalence estimates were extracted. In studies where gender disaggregated prevalence data was not reported, the authors were contacted to request this information.

### Quality appraisal

The quality of the included studies was appraised using a modified Newcastle–Ottawa Scale (see Online Resource 3). The original scale included items on selection bias, comparability between groups and measurement of outcomes, and was modified to include criterion on whether the outcome data was disaggregated by gender, and to be more applicable to cross-sectional studies [7, 8]. The maximum total quality score was 13. Papers were defined as low-quality if they reached a total score between 0 and 4; fair quality if they reached a total score between 5 and 9; and high-quality if they reached a total score between 10 and 13. A second reviewer (SP) independently appraised 20% of the included studies and any disagreements were discussed and resolved.

### Data analysis

Raw data on the prevalence of past year and adult lifetime sexual violence victimisation was extracted from all papers and estimated overall effect sizes and 95% confidence intervals were calculated. A random effects meta-analyses was conducted for past year and adult lifetime sexual violence by gender where possible across all mental health settings i.e. inpatients, outpatients and mixed services where a minimum of three studies were present. Heterogeneity between studies was estimated using the I^2^ statistic. Studies where the data was not disaggregated by gender were excluded from the meta-analyses. Meta-analyses were run for each group, firstly with all studies included and then again with low-quality studies excluded. The results of the meta-analyses are displayed as forest plots.

In papers utilising a control group odds ratios and 95% confidence intervals were extracted to indicate the risk of sexual violence victimisation. In papers where odds ratios were not reported, they were calculated.

We had initially planned to assess the risk of publication bias using a funnel plot. However, due to the small number of papers that included a control group (n = 7), a funnel plot was determined to be inappropriate.

## Results

**Fig. 1:**
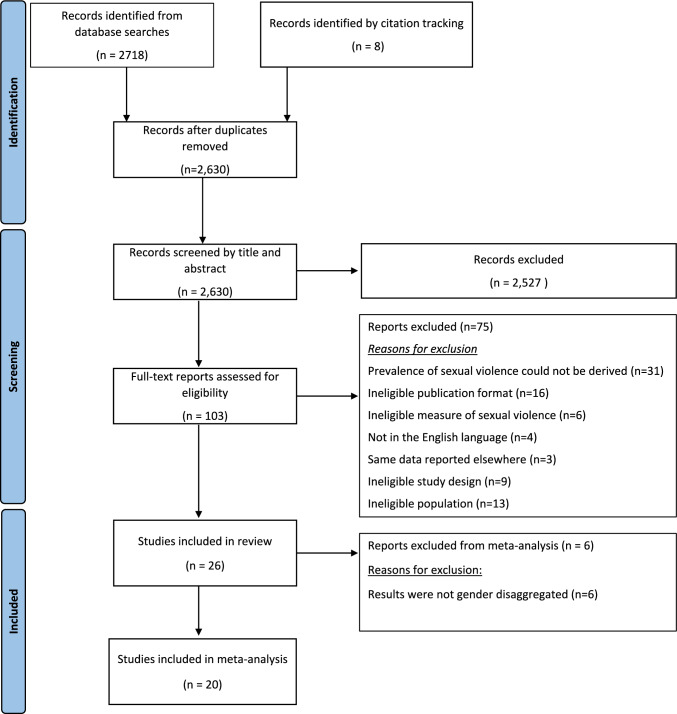
PRISMA Flow diagram of screening and study selection process

The complete study selection process is shown in Fig. 1. The initial database search identified 2,718 records. After de-duplication 2630 titles and abstracts were screened, of which 2527 were excluded. One hundred and three full text papers were screened resulting in the further exclusion of 75 papers. A further 8 papers were identified through citation tracking, resulting in a total of 26 papers to be included in the review. Six of these studies did not present gender disaggregated prevalence data [9–14] and this information could not be obtained from the authors upon contact. This left a total of 20 studies [15–34] which were included in the meta-analyses.

### Study characteristics

The key characteristics of the 26 included studies are presented in Online Resource 4. The included studies spanned publication dates between 1989 and 2020. Four studies were retrospective cohort studies and 22 were cross-sectional studies. Twenty-three papers were conducted in high income countries, of which 8 were from the USA; 4 were from the Netherlands; 2 were from New Zealand, Australia and the UK; and 1 paper was from each of Sweden, France, Greece, Canada and Spain. There were only 3 papers from low-and-middle income countries, of which 2 were from India and 1 was from Brazil. Twenty studies reported their prevalence results separately for men and women whilst gender-disaggregated data could not be obtained for 6 studies.

Fourteen papers measured the prevalence of adult lifetime sexual violence, 6 papers measured the prevalence of past year sexual violence victimisation and 4 papers investigated both. The sample population was comprised of inpatients in 7 papers, outpatients in 12 papers, and a mix of both in 7 papers. The sample population was comprised of male-only participants in 2 papers, female-only participants in 5 papers and both male and female participants in 19 papers.

Some studies recruited participants from specific sub-populations of psychiatric service users, including two studies focusing on veterans and one which focused on episodically homeless women. Several studies used populations with specific diagnoses. Of these, 2 focused on patients with schizophrenia and/or bipolar disorder; 2 looked at patients with psychosis, one focused on patients with a dual diagnosis and one focused on patients with depression. Six studies focused entirely on patients with severe mental illness (SMI) diagnoses.

Control groups were reported in 7 of the 26 included studies. Of these control groups, 5 were derived from the general population, 1 was a medical and surgical patient population, and 1 were relatives of the psychiatric patient sample.

At the quality appraisal stage, 6 high quality papers, 19 fair quality papers and 2 low-quality papers were identified. The results of the quality appraisal are reported in the supporting information (see Online Resource 5).

#### Past year prevalence of sexual violence victimisation by patient setting

The prevalence of sexual violence in the 11 included studies measuring past year sexual violence for male, female and non-gender disaggregated samples in outpatient, inpatient and mixed service settings are presented in Online Resource 6.

### All settings: women

**Fig. 2:**
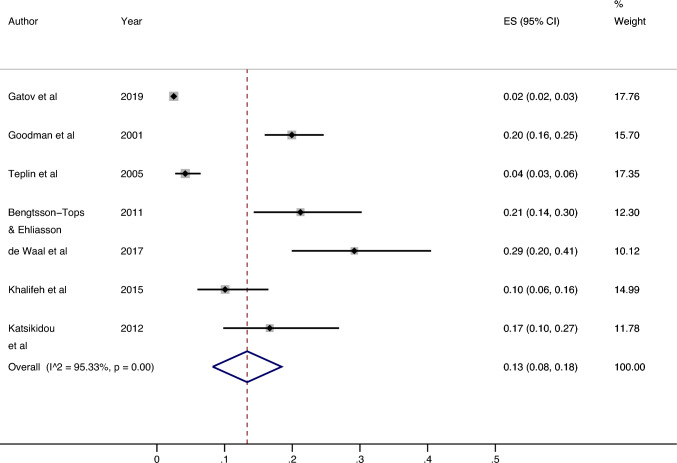
Prevalence of past year sexual violence victimisation among female psychiatric patient populations

Seven studies [14, 15, 21, 22, 24, 27] investigated the past year prevalence of sexual violence in women (see Fig. 2). The lowest prevalence reported was 2.49% (95% CI 2.38%, 2.60%) from a Canadian population-based study utilising data from health-administrative databases [22]. The highest prevalence was 29.17% (95% CI 19.94%, 40.51%) from a Swedish study looking at psychiatric patients with a dual diagnosis [21]. The median prevalence was 16.67% (IQR: 7.14—20.58). The results of the meta-analysis found an estimated overall prevalence of 13.33% (95% CI 8.24%, 18.43%). Heterogeneity between studies was high (I^2^ = 95.33%, p =  < 0.01).

### Outpatients: women

Six papers [14, 15, 21, 22, 26, 27] investigated past year sexual violence victimisation in female outpatients (see Online Resource 7). The lowest prevalence was 2.49% (95% CI 2.38%, 2.60%) [22] and the highest was 29.17% (95% CI 19.94%, 40.51%) [21]. The median prevalence was 13.38% (IQR: 5.66–20.08%). The meta-analysis revealed an estimated overall prevalence of 11.23% (95% CI 6.76%, 15.69%). Heterogeneity between studies was high (I^2^ = 92.57%, p =  < 0.01).

### Inpatients: women

No studies reported the prevalence of past year sexual violence victimisation in female inpatient populations.

### Mixed setting: women

One study reported the prevalence of past year sexual violence victimisation in women from mixed service settings [24]. The prevalence was 19.94% (95% CI 15.93%, 24.65%) in a sample of 782 women with SMI across 4 states in America.

### All settings: Men

**Fig. 3:**
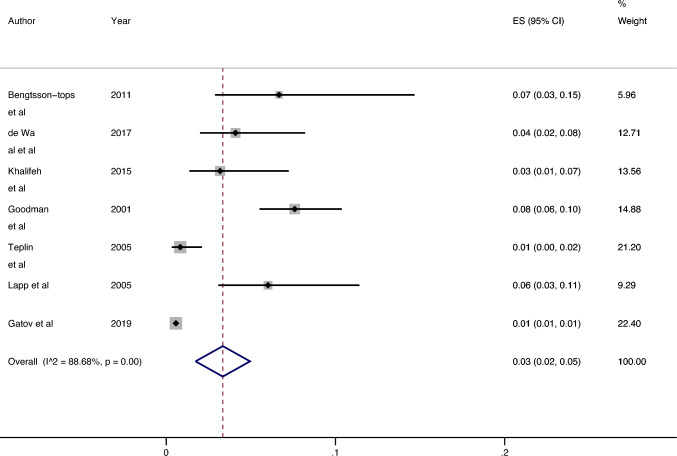
Prevalence of past year sexual violence victimisation among male psychiatric patient populations

Seven studies investigated the past year prevalence of sexual violence victimisation among male psychiatric service users (see Fig. 3). The lowest prevalence was 0.57% (95% CI 0.52–0.63%) [22], and the highest prevalence was 7.59% (95% CI 5.51%, 10.38%) [24]. The median prevalence was 4.09% (IQR: 2.01%, 6.35%). The results of the meta-analysis revealed an estimated overall prevalence of 3.34% (95% CI 1.73%, 4.95%). Heterogeneity between studies was high (I^2^ = 88.68%, p =  < 0.01).

### Outpatients: men

Three papers [14, 15, 21, 22, 27] reported the prevalence of past year prevalence of sexual violence victimisation among male outpatients (see Online Resource 8). The lowest prevalence was 0.57% (95% CI 0.52%, 0.63%) [22] from the Canadian population-based study, and the highest prevalence was 6.67% (95% CI 2.88%, 14.68%) [15] from a Swedish study investigating rates of victimisation in patients with psychosis. The median prevalence was 3.18% (IQR: 0.83–4.09%). The estimated overall prevalence was 1.49% (95% CI 0.43%, 2.56%). Heterogeneity was high between studies (I^2^ = 70.83%, p = 0.01).

### Inpatients: men

One study [28] measured the prevalence of sexual violence in the male inpatient population. The study found a prevalence of 6.02% (95% CI 3.08%, 11.42%) in a sample of male veterans with SMI.

### Mixed settings: men

One study [24] reported the prevalence of past year sexual violence victimisation in men in mixed service settings. They found a prevalence of 7.59% (95% CI 5.51%, 10.38%) in their patients with SMI.

### Studies that were not gender-disaggregated

Three studies reported the prevalence of sexual violence victimisation in psychiatric service users but did not disaggregate their results by gender. This included a Dutch study assessing victimisation in patients with SMI which found that 4.49% (95% CI 1.76%, 10.99%) of their outpatient sample and 13.73% (95% CI 6.81%, 25.72%) of their inpatient sample had experienced sexual violence [10]. The second study found that 1.75% (95% CI 0.80%, 3.76%) out of 9,135 patients with a psychotic disorder had experienced sexual violence [11]. The third study compared the prevalence of sexual violence in three sample populations: the general Amsterdam population; patients in remission from Major Depressive Episodes; and patients with recurrent depression [9]. Since the two patient populations were recruited using different strategies, only the data for the currently depressed patient sample was selected for inclusion in this review.

#### Adult lifetime prevalence of sexual violence victimisation by patient setting

The prevalence of sexual violence in the 14 included studies measuring adult lifetime sexual violence for male, female and non-gender disaggregated samples in outpatient, inpatient and mixed service settings is reported in Online Resource 9.

### All settings: women

**Fig. 4:**
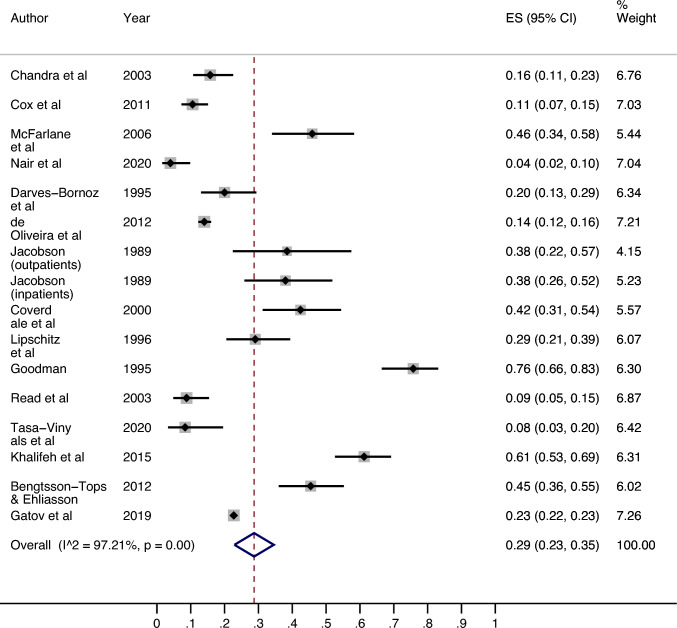
Prevalence of adult lifetime sexual violence victimisation among female psychiatric patient populations

Of the 14 studies measuring the prevalence of adult lifetime sexual violence in female psychiatric service users (see Fig. 4), the lowest prevalence was 4.0% (95% CI 1.57%, 9.84%) [32] in a sample of women with SMI, and highest was 75.76% (95% CI 66.46%, 83.13%) from a sample of episodically homeless women with SMI [23]. The median prevalence was 25.89% (IQR: 13.15%-43.18%). The results of the meta-analysis revealed an estimated overall prevalence of 28.73% (95% CI 22.91%, 34.55%). Heterogeneity between studies was high (I^2^ = 97.21%, p =  < 0.01).

When the 1 low-quality study [33] was excluded from the meta-analysis, there were negligible changes in heterogeneity (I^2^ = 97.26%, p =  < 0.01) and the estimated overall prevalence increased to 30.21% (95% CI 24.33%, 36.29%).

### Outpatients: women

Nine papers reported the adult lifetime prevalence of sexual violence victimisation in female outpatients (see Online Resource 10). Since one study [25] reported separate data for inpatients and outpatients, the outpatient population only was included in this analysis. The lowest prevalence reported was 8.33% (95% CI 3.29%, 19.55%) [34] and the highest prevalence was 75.76% (66.46%, 83.13%) [23]. The median prevalence was 38.46% (IQR: 22.71–45.45%). The meta-analysis found an estimated overall prevalence of 36.07% (95% CI 23.84%, 48.30%). Heterogeneity was high between studies (I^2^ = 97.85%, p =  < 0.01).

When the low-quality study [33] was excluded from the meta-analysis, there was little change in heterogeneity (I– = 97.50%, p =  < 0.01) and the estimated overall prevalence increased to 40.35% (95% CI 25.12%, 56.43%).

### Inpatients: women

Four studies [16, 18, 25, 30, 32] measured the prevalence of sexual violence in female inpatients (see Online Resource 11). The lowest prevalence was 4.0% (95% CI 1.57%, 9.84%) in an Indian sample of women with SMI [32] and the highest prevalence was 45.90% (95% CI 34.01%, 58.28%) in an Australian group of severely mentally ill patients [30]. The median prevalence was 15.75% (IQR: 10.55%-38.0%). The meta-analysis found high heterogeneity between studies (I^2^ = 93.41%, p =  < 0.01) and an estimated overall prevalence of 20.92% (95% CI 10.39%, 31.44%).

### Mixed settings: women

Two papers measured adult lifetime sexual violence victimisation in mixed service settings. The lowest prevalence was 14.02% (95% CI 12.22%, 16.03%) [20] from a Brazilian study investigating the self-report of sexual violence in psychiatric service users. The study with the highest prevalence found that 20.0% (95% CI 13.04%, 29.41%) of patients with schizophrenia or bipolar disorder had experienced sexual violence [19]. The median prevalence was 17.01% (IQR: 15.52–18.51%).

### All settings: men

**Fig. 5:**
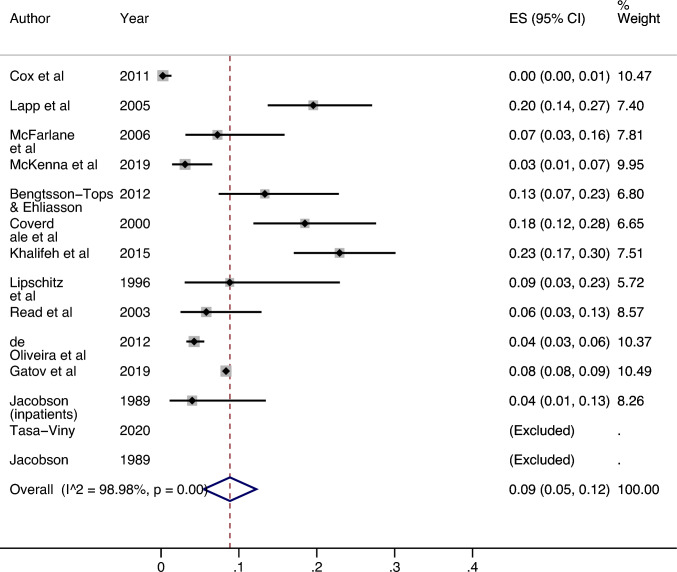
Prevalence of adult lifetime sexual violence victimisation among male psychiatric patient populations

Thirteen studies investigated the adult lifetime prevalence of sexual violence victimisation in male psychiatric service users (see Fig. 5). Two studies reported that 0% of male patients had experienced sexual violence [25, 34]. The highest prevalence was 22.93% (95% CI 17.05%, 30.11%) [27]. The meta-analysis found that heterogeneity between studies was high (I^2^ = 98.98%, p =  < 0.01). The estimated overall prevalence was 8.85% (95% CI 5.47%, 12.23%).

When the low-quality study [33] was excluded from the meta-analysis, heterogeneity increased slightly (I^2^ = 99.07%, p =  < 0.01) and the overall estimated prevalence increased to 9.15% (95% CI 6.43%, 13.21%).

### Outpatients: men

Eight studies measured the prevalence of adult lifetime sexual violence victimisation in male outpatients (see Online Resource 12). The lowest prevalence was 0% from two studies [25; 34] and the highest prevalence was 22.93% (95% CI 17.05%, 30.11%) [27]. The median prevalence was 8.59% (IQR: 4.36–14.62%). The results of the meta-analysis found an estimated overall prevalence of 12.53% (95% CI 7.59%, 17.47%). Heterogeneity was high (I^2^ = 81.98%, p =  < 0.01).

When the low-quality study [33] was removed from the analysis, heterogeneity increased (I^2^ = 85.03%, p =  < 0.01). The estimated overall prevalence remained at 14.22% (95% CI 8.24%, 21.15%).

### Inpatients: men

Five studies reported prevalence data for male inpatients (see Online Resource 13). The lowest prevalence was 0.24% (95% CI 0.04%, 1.34%)[16] in an Indian study investigating rates of sexual coercion, and the highest prevalence was 19.55% (95% CI 13.70%, 27.10%) in sample of veterans with SMI [28]. The median prevalence was 4.0% (IQR: 3.09–7.25%). The meta-analysis revealed an estimated overall prevalence of 5.89% (95% CI 1.41%, 10.37%). Heterogeneity was high (I^2^ = 90.59%, p =  < 0.01).

### Mixed setting: men

One study reported the prevalence of sexual violence in a mixed service setting [20]. They found that 4.26% (95% CI 3.25%, 5.55%) of 1,198 Brazilian psychiatric service users had been sexually victimised.

### Studies with non-gender disaggregated samples

Two studies [12, 13] investigated the adult lifetime prevalence of sexual violence in psychiatric service users but did not disaggregate data by gender. The first study extracted data from the medical records of US military service members admitted to a psychiatric inpatient service for suicide ideation or attempt [12]. They found that participants experienced sexual violence in 11.7% of those admitted for suicide ideation and 20.7% of those admitted for suicide attempt. The combined prevalence across both groups is reported in Online Resource 9. The second study conducted in Australia reported the prevalence of rape in patients who had been assigned a community treatment order (CTO) and those who had not been assigned one [13]. A CTO is an order given by a clinician to provide supervised community treatment. Rape victimisation was reported in 4.5% of the CTO cohort and 3.5% in the non-CTO cohort. The combined prevalence for these two groups is reported in Online Resource 9.

#### Overall risk of past year and adult lifetime sexual violence victimisation among men and women

The odds ratios of past year and lifetime sexual violence victimisation among men and women is shown in Online Resource 14.

Odds ratios for past year victimisation in women are reported in 2 studies [26, 27]. Both found that female psychiatric service users were twice as likely to experience sexual violence than the general population [27] and relatives of medical and surgical outpatients [26]. No odds ratios were reported or calculable for past year sexual violence in men. Four studies reported odds ratios ranging from 2.03 (95% CI 0.94%, 4.41%) to 17.2 (95% CI 10.40%, 28.50%) in non-gender disaggregated samples when comparing past year sexual violence in psychiatric service users to the general population [9–11, 14].

Two studies reported odds ratios for sexual violence across the adult lifetime in men and women separately. Male psychiatric service users were over 2 and 5 times more likely to experience sexual violence than medical and surgical outpatient controls [17] and the general population respectively [27]. The former study found a weaker association in their female sample (OR 1.58; 95% CI 0.77, 3.22) [17] and the latter found that female psychiatric service users were over 4 times more likely to experience sexual violence than the general population [27].

## Discussion

Studies consistently showed a high prevalence of past year and adult lifetime sexual violence victimisation in psychiatric service users, with higher rates found in women than men. Both male and female psychiatric service users were found to have an increased risk of experiencing sexual violence compared to controls. For example, the largest study [14] found their sample of 936 psychiatric service users were over 17 times more likely to experience sexual violence than the 32,449 general population controls. Weaker associations were only found in two studies reporting ORs of 1.17 (95% CI 0.51%, 2.67%) [11] and 1.58 (95% CI 0.77%, 3.22%) [17]. In the former, the authors noted finding a lower prevalence of sexual violence than expected possibly due to the rural study location exhibiting a low general crime rate and a predominantly male sample with a mean age of 49.5 years. The latter study excluded patients with a history of alcohol and substance misuse which are known risk factors for sexual violence victimisation [35] and chose not to approach patients if the subject matter was deemed too sensitive, possibly biasing results.

### Strengths and limitations

This review provides up-to-date estimates on the prevalence of sexual violence in psychiatric service users. Our findings are consistent with other older reviews investigating rates of sexual violence in different populations of people with mental illness [36, 37]. However, this review has several limitations.

The amount of data included in this review may have been limited due to our exclusion of grey literature and papers not written in the English language. Data in our gendered meta-analyses may have been limited as 4 studies did not disaggregate their data by gender and authors either did not respond to requests for this information or informed us it was not retrievable.

The further paucity of data in the included studies meant it was not possible to (a) calculate the pooled odds ratio of sexual violence victimisation for male or female psychiatric service users (b) adjust odds ratios for known confounders e.g., age, ethnicity, income level, substance abuse (c) perform a meta-aggression analysis to account for known confounders. We were also unable to report the prevalence of sexual violence by mental disorder diagnoses as several studies included an unstratified sample of psychiatric service users with a range of different diagnoses, or a group of diagnoses such as SMI which encompassed multiple mental disorders and were defined differently between studies. The small number of included studies resulted in wide confidence intervals for some pooled prevalence estimates, and meant we were unable to devise a funnel plot to assess publication bias.

The distinction between past year and adult lifetime sexual violence is important when presenting sexual violence data. However, participants may not disclose occurrences of past year (or indeed in many cases adult lifetime) sexual violence due insufficient time to process or identify their experiences as non-consensual or abusive. Equally, the measure of adult lifetime sexual violence may introduce recall bias [38]. In general, however, sexual violence is highly underreported by victims due to social stigma, shame, and fears of disbelief [39–41]. Additionally, many of the sexual violence measures utilised in the included studies lack the detail or nuance to reliably identify different forms of sexual violence, which may result in lower prevalence estimates being captured.

The meta-analyses revealed high levels of heterogeneity between studies, even upon exclusion of low-quality studies, possibly due to methodological variations in the primary studies. For instance, studies investigated different sub-groups (e.g. homeless women, male veterans); differently excluded patients with certain mental disorders (e.g. substance abuse, chronic schizophrenia); and had varied requirements around the patients’ level of psychiatric service contact which ranged from 2 days to 2 years. Sample sizes varied widely (*n* = 90–160,436) and 50% of studies had high non-response rates (< 70%). Selection bias may further be introduced in studies where participants were approached by gatekeepers such as case managers, therapists or clinicians.

Comparability is further limited by the range of methods and instruments used to measure sexual violence, which may differentially capture the prevalence of sexual violence across studies. Studies differently used selected sections of a validated questionnaire, a combination of multiple questionnaires or a review of medical charts. The latter is particularly problematic since many clinicians do not routinely ask patients about violence and abuse and therefore medical notes will not capture reliable rates of sexual violence [42]. Furthermore, 50% of studies did not explicitly define the sexual violence outcome being measured, employing nebulous terms such as “sexual assault” without explanation of what experiences this encompassed. Some studies also only measured specific typologies of sexual violence such as rape or sexual coercion. Clarity around the definition of these outcomes is important given that different cultural concepts of sexual violence may affect how participants respond to questioning. Many studies used chart information to ascertain participant mental health diagnoses which will be differently ascribed depending on the clinician, hospital and country they operate in.

### Implications of findings

This review demonstrates key areas for improvement within research and practice. Our findings emphasise the need for a comprehensive and consistent measurement framework for sexual violence to enable reliable and comparable prevalence data to be collected [43]. Studies should ensure all data on sexual violence is gender-disaggregated and measures of association should adjust for social and genetic confounders to allow a more reliable investigation into how sexual violence operates in society [44]. Future studies may address the dearth of research on past year sexual violence in psychiatric inpatients, and from low-middle income countries where the prevalence of sexual violence is often higher [45]. Our findings justify the development of tailored interventions for survivors with psychiatric disorders.

We further emphasise the need for healthcare practitioners to effectively recognise and respond to sexual violence in psychiatric services given their increased risk compared to the general population. Trial interventions have shown that healthcare practitioners can increase the rate of disclosures about violence and abuse through training and the development of clear referral pathways [46–48]. Sexual violence is rooted in a range of social and health determinants, and therefore unified coordination between different systems (healthcare, justice, advocates, social services etc.) is needed to meet the needs of the survivor [49].

### Supplementary Information

Below is the link to the electronic supplementary material.

Supplementary file1 (DOCX 37 KB)

Supplementary file2 (DOCX 22 KB)

Supplementary file3 (DOC 74 KB)

Supplementary file4 (DOCX 52 KB)

Supplementary file5 (DOCX 30 KB)

Supplementary file6 (DOCX 23 KB)

Supplementary file7 (DOCX 82 KB)

Supplementary file8 (DOCX 69 KB)

Supplementary file9 (DOCX 24 KB)

Supplementary file10 (DOCX 117 KB)

Supplementary file11 (DOCX 76 KB)

Supplementary file12 (DOCX 98 KB)

Supplementary file13 (DOCX 70 KB)

Supplementary file14 (DOCX 23 KB)

## Data Availability

No new data was created or analysed in this study.
