# Supplementary material for: Prevalence and risk of sexual violence victimization among mental health service users: a systematic review and meta-analyses
Source: Soc Psychiatry Psychiatr Epidemiol. 2024 Apr 3;59(8):1285–97. doi: 10.1007/s00127-024-02656-8 (PMC11291586; doi:10.1007/s00127-024-02656-8)
Supplement: Supplementary file 3 — Supplementary file3 (DOC 74 KB) [file 127_2024_2656_MOESM3_ESM.doc]

# Online Resource 3: Modified Newcastle Ottawa Quality Appraisal Form

**Article title:** Prevalence and risk of sexual violence victimization among mental health service users: A systematic review and meta-analyses

**Journal name**: Social Psychiatry and Psychiatric Epidemiology

**Author names and affiliations:**

1. **Anjuli Kaul**: Institute of Psychiatry, Psychology & Neuroscience, King’s College London, Health Service and Population Research Department, London, United Kingdom. ORCID ID: 0000-0002-5637-5536
2. **Laura Connell-Jones**: Institute of Psychiatry, Psychology & Neuroscience, King’s College London, Health Service and Population Research Department, London, United Kingdom.
3. **Sharli Anne Paphitis**: Institute of Psychiatry, Psychology & Neuroscience, King’s College London, Health Service and Population Research Department, London, United Kingdom. ORCID ID: 0000-0002-7625-9057
4. **Sian Oram**: Institute of Psychiatry, Psychology & Neuroscience, King’s College London, Health Service and Population Research Department, London, United Kingdom. ORCID ID: 0000-0001-8704-0379

**Corresponding author:** Anjuli Kaul, Institute of Psychiatry, Psychology & Neuroscience at King’s College London, De Crespigny Park, London SE5 8AF, United Kingdom. Email: [anjuli.1.kaul@kcl.ac.uk](mailto:anjuli.1.kaul@kcl.ac.uk).

***Online Resource 3:***

Quality Appraisal Form – Modified Newcastle Ottawa Scale

Name of assessor:

Name of paper:

| Selection (maximum 5 stars) | | | |
| --- | --- | --- | --- |
|  | Question | Comments | Number of stars given |
| 1 | **Representativeness of the sample**   1. **The sample is truly representative of the target population. (2 stars)**   *Patients would be representative if:*   - *Entire source population is represented* - *An unselected sample of consecutive patients are represented* - *A random sample is represented*  1. **Somewhat representative of the average in the target population. (1 star)**  - *Non-random sampling methods were used*  1. **Selected group of users (0 stars)**  - *The researchers purposely selected individuals*  1. **No description of the sampling strategy (0 stars)** |  | *Max. 2 stars* |
| 2 | **Sample size**   - 1. **Justified and satisfactory. (1 star)**   2. **Not justified or satisfactory (0 stars)** |  | *Max. 1 star* |
| 3 | **Non-respondents**   - 1. **The response rate is satisfactory and described**. **(1 star)** - *The non****-****response rate is 70% or above*   1. **The response rate is not described. (0 stars)** - *The non****-****response rate is less than 70%*    1. **No description of the response rate. (0 stars)** |  | *Max. 1 star* |
| 4 | **Ascertainment of the exposure**   - 1. **A validated measurement tool is used to assess mental health (2 stars)**   *Validated measurement tools are defined as*   - *Medical records* - *Structured interviews with clinicians*   1. **Non-validated measurement tools were used to assess mental health, but the tool is available and described (1 star)**   2. **Self-report of mental health (0 stars)**   3. **No description of the measurement tool (0 stars)** |  | *Max. 2 stars* |
| Comparability (max . 1 star) | | | |
| 1 | **If a control group was included, the sample was taken from an appropriate and justified population**   1. **The control groups were taken from both the general population and a clinical population separately (3 stars)** 2. **The control group was taken from the general population only (2 stars)** 3. **The control group was taken from a clinical population only (1 star)** 4. **The control group population was not clearly described (0 stars)** 5. **There was no control group – write N/A** |  | *Max. 3 stars* |
| 2. | **The subjects in different outcome groups are comparable and clearly described, based on the study design or analysis**   - 1. **The study controls for sex (1 star)** - *The patient data (and control group data if applicable) is segregated by sex.*   1. **The study does not control for sex. (0 stars)** - *The patient data (and control group data if applicable) is not segregated by sex* |  | *Max. 1 star* |
| Outcome (Max. 4 stars) | | | |
| 1 | **Definition of outcome**  **Are the main outcomes to be measured clearly described and defined?**   1. **Yes (1 star)** 2. **No (0 stars)** |  | *Max. 1 star* |
| 2 | **Assessment of the outcome**   - 1. **Independent assessment. (2 stars)** - *Information on the experience of sexual violence was obtained through a structured interview.*   1. **Validated patient self-report. (2 stars)** - *The self-report tool is reliable, structured and clearly described*   1. **Record linkage. (1 stars)** - *Information on experience of sexual violence obtained through chart review.*   1. **Non-validated patient self-report. (1 star)** - *The self-report tool used is not reliable, structured, or clearly described.*   1. **No description (0 stars)** |  | *Max. 2 stars* |
| 3 | **Statistical test**   - - - 1. **The statistical test used to analyze the data is clearly described and appropriate, and the measurement of the association is presented, including confidence intervals and the probability level (p value) if applicable. (1 star)**       2. **The statistical test is not appropriate, not described or incomplete. (0 stars)** |  | *Max. 1 star* |

Calculate total score across all 3 sections (out of a possible total of 13)
