# Supplementary material for: Prevalence and risk of sexual violence victimization among mental health service users: a systematic review and meta-analyses
Source: Soc Psychiatry Psychiatr Epidemiol. 2024 Apr 3;59(8):1285–97. doi: 10.1007/s00127-024-02656-8 (PMC11291586; doi:10.1007/s00127-024-02656-8)
Supplement: Supplementary file 4 — Supplementary file4 (DOCX 52 KB) [file 127_2024_2656_MOESM4_ESM.docx]

# Online Resource 4: Table of characteristics of included studies

**Article title:** Prevalence and risk of sexual violence victimization among mental health service users: A systematic review and meta-analyses

**Journal name**: Social Psychiatry and Psychiatric Epidemiology

**Author names and affiliations:**

1. **Anjuli Kaul**: Institute of Psychiatry, Psychology & Neuroscience, King’s College London, Health Service and Population Research Department, London, United Kingdom. ORCID ID: 0000-0002-5637-5536
2. **Laura Connell-Jones**: Institute of Psychiatry, Psychology & Neuroscience, King’s College London, Health Service and Population Research Department, London, United Kingdom.
3. **Sharli Anne Paphitis**: Institute of Psychiatry, Psychology & Neuroscience, King’s College London, Health Service and Population Research Department, London, United Kingdom. ORCID ID: 0000-0002-7625-9057
4. **Sian Oram**: Institute of Psychiatry, Psychology & Neuroscience, King’s College London, Health Service and Population Research Department, London, United Kingdom. ORCID ID: 0000-0001-8704-0379

**Corresponding author:** Anjuli Kaul, Institute of Psychiatry, Psychology & Neuroscience at King’s College London, De Crespigny Park, London SE5 8AF, United Kingdom. Email: [anjuli.1.kaul@kcl.ac.uk](mailto:anjuli.1.kaul@kcl.ac.uk).

**Online Resource 4: Table of characteristics of included studies**

| **Author and year** | **Country** | **Setting** | **Study Design** | **Patient inclusion criteria** | **Type of outcome** | **Mental Health Assessment** | **Violence Assessment** | **Patient sample size (n)** | **Control group characteristics** | **Quality appraisal score (max. =13)** |
| --- | --- | --- | --- | --- | --- | --- | --- | --- | --- | --- |
| Bengtsson-Tops & Ehliasson, 2012 | Sweden | Outpatients | Cross-sectional | Psychosis diagnosis, living in the community, ongoing service contact, ability to give informed consent | Adult lifetime and past year sexual violence | Chart diagnosis | Structured questionnaire including the Composite Abuse Scale and sections of the Bengtsson-Tops question manual | 174 | No control group | 7 |
| Chandra et al, 2003 | India | Inpatients | Cross-sectional | Hospital stay of >1 week, ability to be interviewed | Adult lifetime sexual violence | ICD-10 | The Sexual Experiences Survey | 146 | No control group | 9 |
| Christ et al, 2018 | Netherlands | Outpatients | Cross-sectional | Primary diagnosis of any depressive disorder, ≥ 17 years old, Dutch speaking | Past year sexual violence | The Inventory of Depressive Symptomatology – Self Report (IDS-SR; Dutch version) | Section 4 of the Safety Monitor | 102 | General population of Amsterdam, age 18-65 years. *n* =9,175 | 8 |
| Coverdale et al, 2000 | New Zealand | Outpatients | Cross-sectional | Excluded patients with primary diagnosis of "mental retardation" or alcohol/substance misuse. Did not approach patients if subject matter deemed too sensitive. | Adult lifetime sexual violence | Chart diagnosis and consultation with Community Mental Health Center (CMHC) staff | Structured questionnaire based on previous studies on psychiatric patient populations | 158 | Medical and surgical outpatients matched to patient group for ethnicity and age. Included if never been seen by a psychiatrist/psychologist or treated for psychiatric illness. *n* =158 | 6 |
| Cox et al, 2011 | USA | Inpatients | Retrospective chart review | Veterans, admission due to suicide-related thoughts/behaviours. | Adult lifetime sexual violence | Chart diagnosis | Chart review | 656 | No control group | 9 |
| Darves-Bornoz et al, 1995 | France | Mixed | Cross-sectional | Female, schizophrenia or bipolar disorder diagnosis, aged 18-45 years | Adult lifetime sexual violence | DSM-III-R | Semi-structured interview given by a psychiatrist | 90 | No control group | 8 |
| de Mooij, 2015 | The Netherlands | Mixed | Cross-sectional | SMI (DSM-IV diagnosis of schizophrenia, a psychotic disorder, substance use disorder, severe mood or anxiety disorder), 2-year history of continuous intensive mental healthcare, patients with dual diagnosis, English/Dutch speaking, living in Amsterdam for ≤1year, excluded if too ill to participate or impaired communication. | Past year sexual violence | BPRS-E | Dutch version of The Integral Safety/Security Monitor | 323 | General population of Amsterdam. N=10,865 | 9 |
| de Oliveira et al, 2012 | Brazil | Mixed | Cross-sectional | ≥ 18 years, able to be interviewed | Adult lifetime sexual violence | ICD-10 | Self-reported/semi-structured interviews | 2,475 | No control group | 10 |
| de Vries et al, 2019 | Netherlands | Mixed | Cross-sectional | Using antipsychotic medication, ≥ 18 years, diagnosis in psychotic spectrum (DSM-IV). | Past year sexual violence | DSM-IV | Dutch crime and victimization survey (IVM) | 343 | General population in same provinces as patient group. N=9,135 | 9 |
| de Waal et al, 2017 | Netherlands | Outpatients | Cross-sectional | ≥ 18 years, DSM-IV substance abuse/dependence diagnoses plus at least one other DSM-IV Axis I or II mental disorder, Dutch speaking, eligible for group therapy according to case manager | Past year sexual violence | DSM-IV | The Safety Monitor | 243 | No control group | 6 |
| Gatov et al, 2019 | Canada | Outpatients | Cross-sectional | Ontario residents, aged 18-105 years, admitted between 1st April 2009 - 31st March 2016. Excluded forensic patients, patients with missing discharge/diagnostic information | Past year sexual violence | Chart diagnosis | RAI-MH | 160,436 | No control group | 10 |
| Goodman, 1995 | USA | Outpatients | Cross-sectional | Female, SMI diagnosis, referred from psychiatric hospital local shelter to the service. Formerly homeless (defined as having lived in a shelter/on the street/ no fixed address at point of discharge) | Adult lifetime sexual violence | Diagnosis provided by case manager | Modified version of the Violence Subscale of Straus's (1979) Conflict Tactics scale and Russel's (1986) semi structured interview | 99 | No control group | 6 |
| Goodman et al, 2001 | USA | Mixed | Cross-sectional | SMI (according to definition of the state of each study location), able to give informed consent, age 18-60 years. | Past year sexual violence | DSM-IV and chart diagnosis | Two subscales of the Revised Conflict Tactic Scales | 782 | No control group | 7 |
| Jacobson, 1989 | USA | Mixed | Cross-sectional | New patients, excluded patients with chronic schizophrenia. | Adult lifetime sexual violence | Chart diagnosis | Semi-structured interview | 131 | No control group | 6 |
| Katsikidou et al, 2012 | Greece | Outpatients | Cross-sectional | Previous admission at same hospital, DSM-IV-TR diagnosis of schizophrenia/ schizoaffective disorder/bipolar disorder. Excluded patients with dementia, organic brain damage, primary diagnosis of substance use disorder, acute illness relapse. | Past year sexual violence | DSM-IV-TR | Semi-structured interview based on NCVS and the ICVS | 150 | Relatives of inpatients in the general medical and surgical departments of the same hospital. Matched for gender, age and sociocultural identity. Excluded people who reported current/past psychiatric treatment. N=150 | 11 |
| Khalifeh et al, 2015 | UK | Outpatients | Cross-sectional | Age 18–59 years, under the care of CMHTs in one of six London boroughs for ≥1 year, living in community, capacity to consent, English speaker, capacity to consent. | Both sexual violence | ICD-10 | ONS National Crime Survey | 286 | Participants of the 2011–2012 ONS crime survey (CSEW). One adult per private residential household was recruited. Included if aged 18–59 years, completed the domestic/sexual violence module. Adult lifetime sample: n=22,606; Past year sample n=22,605 | 12 |
| Lapp et al, 2005 | USA | Inpatients | Cross-sectional | ≥ 18 years, veterans with SMI who participated in previous study on HIV prevalence and risk behaviours, admitted between March 1997-June 2000. | Both sexual violence | Chart diagnosis, PTSD Checklist (PCL).17 and DSMIV | Physical and sexual assault subscales of the revised Conflict Tactics Scales | 133 | No control group | 7 |
| Lipschitz et al, 1996 | USA | Outpatients | Cross-sectional | None specified | Adult lifetime sexual violence | DSM-III-R | Traumatic Events Questionnaire | 120 | No control group | 8 |
| McFarlane et al, 2006 | Australia | Inpatients | Cross-sectional | Admitted for ≥ 2 days, English speaking. Excluded if too ill to participate. | Adult lifetime sexual violence | The Posttraumatic Stress Disorder and Substance Abuse sections of the Composite International Diagnostic Interview | Traumatic Antecedents Questionnaire | 130 | No control group | 7 |
| McKenna et al, 2019 | UK | Inpatients | Retrospective chart review | Excluded patients on trial leave. | Adult lifetime sexual violence | Chart diagnosis | Trauma History Questionnaire | 194 | No control group | 10 |
| Nair, 2020 | India | Inpatients | Cross-sectional | Female, age 18–50 years, SMI, have a cohabiting partner. Excluded those with history suggestive of cognitive deficits and intellectual disability. | Adult lifetime and past year sexual violence | BPRS | IFVCS | 100 | No control group | 8 |
| Read et al, 2003 | New Zealand | Outpatients | Retrospective chart review | None specified | Adult lifetime sexual violence | DSM-IV | Chart review | 200 | No control group | 4 |
| Ryan et al, 2020 | USA | Inpatients | Retrospective chart review | ≥ 18 years, admitted between 1st January 2001-31st December 2006. If patient was hospitalised multiple times, only the first hospitalisation was included. | Adult lifetime sexual violence | Chart diagnosis | Chart review | 1100 | No control group | 4 |
| Segal et al, 2019 | Australia | Mixed | Cross-sectional | Obtained a CTO or had service contact between 1st July 2000 - 30th June 2010. | Adult lifetime sexual violence | Clinician CTO evaluations and the independent Health of the Nations Scale (HoNOS) severity-score profile assessments | LEAP-reports | 27,585 | No control group | 8 |
| Tasa-Vinyals et al, 2020 | Spain | Outpatients | Cross-sectional | ≥ 18 year, DSM-IV-R diagnosis of SMD (schizophrenia, schizoaffective disorder, or bipolar disorder), attended service between 2007–2009. Excluded patients who scored 4 in any items in the Brief Psychotic Rating Scale or ≥ 3 in conceptual disorganization, disorganized and unusual thinking, or auditory hallucinations. | Adult lifetime sexual violence | BPRS and DEQ | TLEQ | 102 | No control group | 6 |
| Teplin et al, 2005 | USA | Outpatients | Cross-sectional | SMI or if on psychiatric medication or previous psychiatric hospitalisation. Excluded patients arriving for their first visit and those receiving crisis management services | Past year sexual violence | CIDI version 2.1 | NCVS | 936 | General population. NCVS data from largest cities in all US standard metropolitan areas. N= 32,449 | 10 |
