# Supplementary material for: Prevalence and risk of sexual violence victimization among mental health service users: a systematic review and meta-analyses
Source: Soc Psychiatry Psychiatr Epidemiol. 2024 Apr 3;59(8):1285–97. doi: 10.1007/s00127-024-02656-8 (PMC11291586; doi:10.1007/s00127-024-02656-8)
Supplement: Supplementary file 5 — Supplementary file5 (DOCX 30 KB) [file 127_2024_2656_MOESM5_ESM.docx]

# Online Resource 5: Table of results of the quality appraisal

**Article title:** Prevalence and risk of sexual violence victimization among mental health service users: A systematic review and meta-analyses

**Journal name**: Social Psychiatry and Psychiatric Epidemiology

**Author names and affiliations:**

1. **Anjuli Kaul**: Institute of Psychiatry, Psychology & Neuroscience, King’s College London, Health Service and Population Research Department, London, United Kingdom. ORCID ID: 0000-0002-5637-5536
2. **Laura Connell-Jones**: Institute of Psychiatry, Psychology & Neuroscience, King’s College London, Health Service and Population Research Department, London, United Kingdom.
3. **Sharli Anne Paphitis**: Institute of Psychiatry, Psychology & Neuroscience, King’s College London, Health Service and Population Research Department, London, United Kingdom. ORCID ID: 0000-0002-7625-9057
4. **Sian Oram**: Institute of Psychiatry, Psychology & Neuroscience, King’s College London, Health Service and Population Research Department, London, United Kingdom. ORCID ID: 0000-0001-8704-0379

**Corresponding author:** Anjuli Kaul, Institute of Psychiatry, Psychology & Neuroscience at King’s College London, De Crespigny Park, London SE5 8AF, United Kingdom. Email: [anjuli.1.kaul@kcl.ac.uk](mailto:anjuli.1.kaul@kcl.ac.uk).

***Online Resource 5: Table of results of the quality appraisal***

| **Study name and year** | **Representativeness of the sample**  **(max. score= 2)** | **Sample size**  **(max. score = 1)** | **Non-respondents**  **(max. score = 1)** | **Ascertainment of the exposure**  **(max. score = 2)** | **If a control group was included, the sample was taken from an appropriate and justified population**  **(max. score = 2)** | **The subjects in different outcome groups are comparable and clearly described, based on the study design or analysis**  **(max. score = 1)** | **Definition of outcome**  **(max. score = 1)** | **Assessment of the outcome**  **(max. score = 2)** | **Statistical test**  **(max. score = 1)** | **Total (max score = 13)** |
| --- | --- | --- | --- | --- | --- | --- | --- | --- | --- | --- |
| Bengtsson-Tops & Ehliasson, 2012 | 1 | 0 | 1 | 2 | N/A | 1 | 0 | 2 | 0 | 7 |
| Chandra et al, 2003 | 2 | 0 | 1 | 2 | N/A | 1 | 1 | 2 | 0 | 9 |
| Christ et al, 2018 | 0 | 1 | 0 | 2 | 2 | 0 | 0 | 2 | 1 | 8 |
| Coverdale et al, 2000 | 1 | 0 | 0 | 2 | 1 | 1 | 1 | 0 | 0 | 6 |
| Cox et al, 2011 | 2 | 1 | 1 | 2 | N/A | 1 | 0 | 1 | 1 | 9 |
| Darves-Bornoz et al, 1995 | 0 | 0 | 1 | 2 | N/A | 1 | 1 | 2 | 1 | 8 |
| de Mooij, 2015 | 2 | 0 | 0 | 2 | 2 | 0 | 0 | 2 | 1 | 9 |
| de Oliveira et al, 2012 | 2 | 1 | 1 | 2 | N/A | 1 | 1 | 2 | 0 | 10 |
| de Vries et al, 2019 | 1 | 1 | 0 | 2 | 2 | 0 | 0 | 2 | 1 | 9 |
| de Waal et al, 2017 | 1 | 0 | 0 | 2 | N/A | 1 | 0 | 2 | 0 | 6 |
| Gatov et al, 2019 | 2 | 1 | 1 | 2 | N/A | 1 | 0 | 2 | 1 | 10 |
| Goodman, 1995 | 1 | 1 | 1 | 0 | N/A | 1 | 1 | 1 | 0 | 6 |
| Goodman et al, 2001 | 0 | 0 | 1 | 2 | N/A | 1 | 1 | 2 | 0 | 7 |
| Jacobson, 1989 | 0 | 0 | 0 | 2 | N/A | 1 | 1 | 2 | 0 | 6 |
| Katsikidou et al, 2012 | 2 | 1 | 1 | 2 | 1 | 1 | 0 | 2 | 1 | 11 |
| Khalifeh et al, 2015 | 2 | 1 | 0 | 2 | 2 | 1 | 1 | 2 | 1 | 12 |
| Lapp et al, 2005 | 0 | 0 | 1 | 2 | N/A | 1 | 1 | 2 | 0 | 7 |
| Lipschitz et al, 1996 | 0 | 0 | 1 | 2 | N/A | 1 | 1 | 2 | 1 | 8 |
| McFarlane et al, 2006 | 0 | 1 | 0 | 2 | N/A | 1 | 0 | 2 | 1 | 7 |
| McKenna et al, 2019 | 2 | 1 | 1 | 2 | N/A | 1 | 1 | 2 | 0 | 10 |
| Nair, 2020 | 1 | 0 | 0 | 2 | N/A | 1 | 1 | 2 | 1 | 8 |
| Read et al, 2003 | 0 | 0 | 0 | 2 | N/A | 1 | 0 | 1 | 0 | 4 |
| Ryan et al, 2020 | 0 | 0 | 0 | 2 | N/A | 0 | 0 | 1 | 1 | 4 |
| Segal et al, 2019 | 2 | 0 | 1 | 2 | N/A | 0 | 0 | 2 | 1 | 8 |
| Tasa-Vinyals et al, 2020 | 0 | 0 | 0 | 2 | N/A | 1 | 1 | 2 | 0 | 6 |
| Teplin et al, 2005 | 2 | 1 | 0 | 2 | 2 | 0 | 0 | 2 | 1 | 10 |
