# Supplementary material for: Prevalence and risk of sexual violence victimization among mental health service users: a systematic review and meta-analyses
Source: Soc Psychiatry Psychiatr Epidemiol. 2024 Apr 3;59(8):1285–97. doi: 10.1007/s00127-024-02656-8 (PMC11291586; doi:10.1007/s00127-024-02656-8)
Supplement: Supplementary file 6 — Supplementary file6 (DOCX 23 KB) [file 127_2024_2656_MOESM6_ESM.docx]

# Online Resource 6: Table showing the past year prevalence of sexual violence victimisation by patient setting

**Article title:** Prevalence and risk of sexual violence victimization among mental health service users: A systematic review and meta-analyses

**Journal name**: Social Psychiatry and Psychiatric Epidemiology

**Author names and affiliations:**

1. **Anjuli Kaul**: Institute of Psychiatry, Psychology & Neuroscience, King’s College London, Health Service and Population Research Department, London, United Kingdom. ORCID ID: 0000-0002-5637-5536
2. **Laura Connell-Jones**: Institute of Psychiatry, Psychology & Neuroscience, King’s College London, Health Service and Population Research Department, London, United Kingdom.
3. **Sharli Anne Paphitis**: Institute of Psychiatry, Psychology & Neuroscience, King’s College London, Health Service and Population Research Department, London, United Kingdom. ORCID ID: 0000-0002-7625-9057
4. **Sian Oram**: Institute of Psychiatry, Psychology & Neuroscience, King’s College London, Health Service and Population Research Department, London, United Kingdom. ORCID ID: 0000-0001-8704-0379

**Corresponding author:** Anjuli Kaul, Institute of Psychiatry, Psychology & Neuroscience at King’s College London, De Crespigny Park, London SE5 8AF, United Kingdom. Email: [anjuli.1.kaul@kcl.ac.uk](mailto:anjuli.1.kaul@kcl.ac.uk).

***Online Resource 6: Table showing the past year prevalence of sexual violence victimisation by patient setting***

| Setting | Author and year | Prevalence in men % (95% CI) | Prevalence in women % (95% CI) | Prevalence in non-gender disaggregated samples % (95% CI) |
| --- | --- | --- | --- | --- |
| Outpatient | Bengtsson-Tops & Ehliasson, 2012 | 6.67 (2.88, 14.68) | 21.21 (14.31, 30.26) |  |
|  | Christ et al, 2018 |  |  | 7.2 (3.36, 13.49) |
|  | de Waal et al, 2017 | 4.09 (2.0, 8.31) | 29.17 (19.94, 40.51) |  |
|  | de Mooij et al, 2015 |  |  | 4.49 (1.76, 10.99) |
|  | Gatov et al, 2019 | 0.57 (0.52, 0.63) | 2.49 (2.38, 2.60) |  |
|  | Katsikidou et al, 2012 |  | 16.67 (9.80, 26.91) |  |
|  | Khalifeh et al, 2015 | 3.18 (1.37, 7.24) | 10.08 (5.98, 16.48) |  |
|  | Teplin et al, 2005 | 0.83 (0.32, 2.11) | 4.19 (2.70, 6.46) |  |
| Inpatient | de Mooij et al, 2015 |  |  | 13.73 (6.81, 25.72) |
|  | Lapp et al, 2005 | 6.02 (3.08, 11.42) |  |  |
| Mixed | de Vries, 2019 |  |  | 1.75 (0.80, 3.76) |
|  | Goodman et al, 2001 | 7.59 (5.51, 10.38) | 19.94 (15.93, 24.65) |  |
