# Supplementary material for: Prevalence and risk of sexual violence victimization among mental health service users: a systematic review and meta-analyses
Source: Soc Psychiatry Psychiatr Epidemiol. 2024 Apr 3;59(8):1285–97. doi: 10.1007/s00127-024-02656-8 (PMC11291586; doi:10.1007/s00127-024-02656-8)
Supplement: Supplementary file 9 — Supplementary file9 (DOCX 24 KB) [file 127_2024_2656_MOESM9_ESM.docx]

# Online Resource 9: Table showing the prevalence of adult lifetime sexual violence victimisation in psychiatric patient populations

**Article title:** Prevalence and risk of sexual violence victimization among mental health service users: A systematic review and meta-analyses

**Journal name**: Social Psychiatry and Psychiatric Epidemiology

**Author names and affiliations:**

1. **Anjuli Kaul**: Institute of Psychiatry, Psychology & Neuroscience, King’s College London, Health Service and Population Research Department, London, United Kingdom. ORCID ID: 0000-0002-5637-5536
2. **Laura Connell-Jones**: Institute of Psychiatry, Psychology & Neuroscience, King’s College London, Health Service and Population Research Department, London, United Kingdom.
3. **Sharli Anne Paphitis**: Institute of Psychiatry, Psychology & Neuroscience, King’s College London, Health Service and Population Research Department, London, United Kingdom. ORCID ID: 0000-0002-7625-9057
4. **Sian Oram**: Institute of Psychiatry, Psychology & Neuroscience, King’s College London, Health Service and Population Research Department, London, United Kingdom. ORCID ID: 0000-0001-8704-0379

**Corresponding author:** Anjuli Kaul, Institute of Psychiatry, Psychology & Neuroscience at King’s College London, De Crespigny Park, London SE5 8AF, United Kingdom. Email: [anjuli.1.kaul@kcl.ac.uk](mailto:anjuli.1.kaul@kcl.ac.uk).

***Online Resource 9: Table showing the prevalence of adult lifetime sexual violence victimisation in psychiatric patient populations***

| **Setting** | **Author and year** | **Prevalence in men % (95% CI)** | **Prevalence in women % (95% CI)** | **Non-gender disaggregated samples % (95% CI)** |
| --- | --- | --- | --- | --- |
| Outpatient | Bengtsson-Tops & Ehliasson, 2012 | 13.33 (7.41, 22.83) | 45.45 (36.00, 55.25) |  |
|  | Coverdale et al, 2000 | 18.48 (11.87, 27.61) | 42.42 (31.24, 54.44) |  |
|  | Gatov et al, 2019 | 8.35 (8.16, 8.54) | 22.71 (22.42, 23.01) |  |
|  | Goodman, 1995 |  | 75.76 (66.46, 83.13) |  |
|  | Jacobson (outpatients), 1989 | 0.00 (0.00, 43.45) | 38.46 (22.43, 57.47) |  |
|  | Khalifeh et al, 2015 | 22.93 (17.05, 30.11) | 61.24 (52.62, 69.21) |  |
|  | Lipschitz et al, 1996 | 8.82 (3.05, 22.96) | 29.07 (20.53, 39.40) |  |
|  | Read et al, 2003 | 5.81 (2.51, 12.90) | 8.77 (4.83, 15.40) |  |
|  | Tasa-Vinyals et al, 2020 | 0.00 (0.00, 6.64) | 8.33 (3.29, 19.55) |  |
| Inpatient | Chandra et al, 2003 |  | 15.75 (10.73, 22.53) |  |
|  | Cox et al, 2011 | 0.24 (0.04, 1.34) | 10.55 (7.25, 15.11) |  |
|  | Jacobson (inpatients), 1989 | 4.00 (1.10, 13.46) | 38.00 (25.86, 51.85) |  |
|  | Lapp et al, 2005 | 19.55 (13.70, 27.10) |  |  |
|  | McKenna et al, 2019 | 3.09 (1.43, 6.58) |  |  |
|  | McFarlane et al, 2006 | 7.25 (3.13, 15.87) | 45.90 (34.01, 58.28) |  |
|  | Nair et al, 2020 |  | 4.00 (1.57, 9.84) |  |
|  | Ryan et al, 2020 |  |  | 4.91 (3.78, 6.35) |
| Mixed setting | Darves-Bornoz et al, 1995 |  | 20.00 (13.04, 29.41) |  |
|  | de Oliveira et al, 2012 | 4.26 (3.25, 5.55) | 14.02 (12.22, 16.03) |  |
|  | Segal et al, 2019 |  |  | 3.89 (3.67, 4.12) |
