# Supplementary material for: Prevalence and risk of sexual violence victimization among mental health service users: a systematic review and meta-analyses
Source: Soc Psychiatry Psychiatr Epidemiol. 2024 Apr 3;59(8):1285–97. doi: 10.1007/s00127-024-02656-8 (PMC11291586; doi:10.1007/s00127-024-02656-8)
Supplement: Supplementary file 10 — Supplementary file10 (DOCX 117 KB) [file 127_2024_2656_MOESM10_ESM.docx]

# Online Resource 10: Prevalence of adult lifetime sexual violence victimisation among female psychiatric outpatient populations

**Article title:** Prevalence and risk of sexual violence victimization among mental health service users: A systematic review and meta-analyses

**Journal name**: Social Psychiatry and Psychiatric Epidemiology

**Author names and affiliations:**

1. **Anjuli Kaul**: Institute of Psychiatry, Psychology & Neuroscience, King’s College London, Health Service and Population Research Department, London, United Kingdom. ORCID ID: 0000-0002-5637-5536
2. **Laura Connell-Jones**: Institute of Psychiatry, Psychology & Neuroscience, King’s College London, Health Service and Population Research Department, London, United Kingdom.
3. **Sharli Anne Paphitis**: Institute of Psychiatry, Psychology & Neuroscience, King’s College London, Health Service and Population Research Department, London, United Kingdom. ORCID ID: 0000-0002-7625-9057
4. **Sian Oram**: Institute of Psychiatry, Psychology & Neuroscience, King’s College London, Health Service and Population Research Department, London, United Kingdom. ORCID ID: 0000-0001-8704-0379

**Corresponding author:** Anjuli Kaul, Institute of Psychiatry, Psychology & Neuroscience at King’s College London, De Crespigny Park, London SE5 8AF, United Kingdom. Email: [anjuli.1.kaul@kcl.ac.uk](mailto:anjuli.1.kaul@kcl.ac.uk).

***Online Resource 10: Prevalence of adult lifetime sexual violence victimisation among female psychiatric outpatient populations***

***
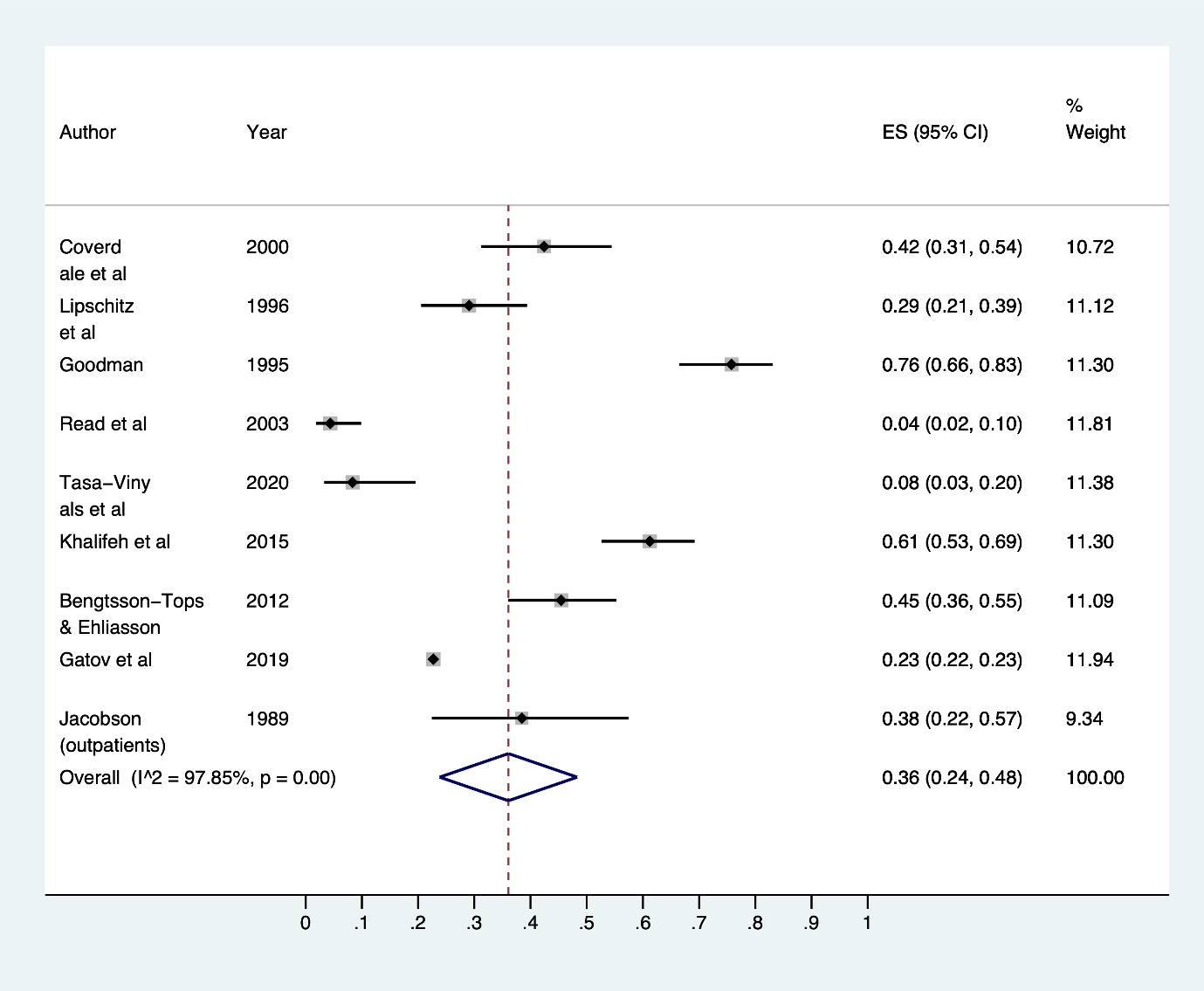
***
