# Supplementary material for: Prevalence and risk of sexual violence victimization among mental health service users: a systematic review and meta-analyses
Source: Soc Psychiatry Psychiatr Epidemiol. 2024 Apr 3;59(8):1285–97. doi: 10.1007/s00127-024-02656-8 (PMC11291586; doi:10.1007/s00127-024-02656-8)
Supplement: Supplementary file 14 — Supplementary file14 (DOCX 23 KB) [file 127_2024_2656_MOESM14_ESM.docx]

# Online Resource 14: Table showing the overall odds of past year and adult lifetime sexual violence victimisation among men and women

**Article title:** Prevalence and risk of sexual violence victimization among mental health service users: A systematic review and meta-analyses

**Journal name**: Social Psychiatry and Psychiatric Epidemiology

**Author names and affiliations:**

1. **Anjuli Kaul**: Institute of Psychiatry, Psychology & Neuroscience, King’s College London, Health Service and Population Research Department, London, United Kingdom. ORCID ID: 0000-0002-5637-5536
2. **Laura Connell-Jones**: Institute of Psychiatry, Psychology & Neuroscience, King’s College London, Health Service and Population Research Department, London, United Kingdom.
3. **Sharli Anne Paphitis**: Institute of Psychiatry, Psychology & Neuroscience, King’s College London, Health Service and Population Research Department, London, United Kingdom. ORCID ID: 0000-0002-7625-9057
4. **Sian Oram**: Institute of Psychiatry, Psychology & Neuroscience, King’s College London, Health Service and Population Research Department, London, United Kingdom. ORCID ID: 0000-0001-8704-0379

**Corresponding author:** Anjuli Kaul, Institute of Psychiatry, Psychology & Neuroscience at King’s College London, De Crespigny Park, London SE5 8AF, United Kingdom. Email: [anjuli.1.kaul@kcl.ac.uk](mailto:anjuli.1.kaul@kcl.ac.uk).

***Online Resource 14: Odds of sexual violence victimisation prevalence in mental health service users compared to controls***

|  | **Setting** | **Author** | **OR Women (95% CI)** | **OR Men (95% CI)** | **OR non gender disaggregated sample (95% CI)** |
| --- | --- | --- | --- | --- | --- |
| **Past year** | Outpatients | Khalifeh et al, 2015 | 2.10 (0.98, 4.70)^b^ | ^a^ |  |
|  |  | Christ et al, 2018 |  |  | 2.03 (0.94, 4.41) |
|  |  | Teplin et al, 2005 |  |  | 17.20 (10.40, 28.50) |
|  |  | Katsikidou et al, 2012 | 2.17 (0.77, 6.14) |  |  |
|  |  | de Mooij, 2015 |  |  | 3.32 (1.20, 9.16) |
|  | Inpatients | de Mooij, 2015 |  |  | 11.21 (4.97, 25.29) |
|  | Mixed | de Vries, 2019 |  |  | 1.17 (0.51, 2.67) |
| **Adult lifetime** | Outpatients | Coverdale et al, 2000 | 1.58 (0.77, 3.22) | 2.75 (1.08, 7.0) |  |
|  |  | Khalifeh et al, 2015 | 4.40 (2.90, 6.80)^b^ | 5.50 (3.20, 9.50)^b^ |  |

*^a^ Study did not calculate an OR because the absolute numbers were considered too low to allow for stable estimates.*

*^b^ Adjusted for age, ethnicity, marital status, living alone, having children, employment, housing tenure, area deprivation, frequency of drunkenness in past year and any past-year illicit drug use.*
